# Anoctamin 5 (ANO5) Muscle Disorders: A Narrative Review

**DOI:** 10.3390/genes13101736

**Published:** 2022-09-27

**Authors:** Pannathat Soontrapa, Teerin Liewluck

**Affiliations:** 1 Division of Neuromuscular Medicine, Department of Neurology, Mayo Clinic, Rochester, MN 55905, USA; 2 Division of Neurology, Department of Medicine, Faculty of Medicine Siriraj Hospital, Mahidol University, Bangkok 10700, Thailand

**Keywords:** ANO5, anoctamin 5, anoctaminopathy, LGMD, LGMDR12, limb–girdle muscular dystrophy, Miyoshi myopathy, MMD3

## Abstract

Anoctaminopathy-5 refers to a group of hereditary skeletal muscle or bone disorders due to mutations in the anoctamin 5 (ANO5)-encoding gene, *ANO5*. ANO5 is a 913-amino acid protein of the anoctamin family that functions predominantly in phospholipid scrambling and plays a key role in the sarcolemmal repairing process. Monoallelic mutations in *ANO5* give rise to an autosomal dominant skeletal dysplastic syndrome (gnathodiaphyseal dysplasia or GDD), while its biallelic mutations underlie a continuum of four autosomal recessive muscle phenotypes: (1). limb–girdle muscular dystrophy type R12 (LGMDR12); (2). Miyoshi distal myopathy type 3 (MMD3); (3). metabolic myopathy-like (pseudometabolic) phenotype; (4). asymptomatic hyperCKemia. ANO5 muscle disorders are rare, but their prevalence is relatively high in northern European populations because of the founder mutation c.191dupA. Weakness is generally asymmetric and begins in proximal muscles in LGMDR12 and in distal muscles in MMD3. Patients with the pseudometabolic or asymptomatic hyperCKemia phenotype have no weakness, but conversion to the LGMDR12 or MMD3 phenotype may occur as the disease progresses. There is no clear genotype–phenotype correlation. Muscle biopsy displays a broad spectrum of pathology, ranging from normal to severe dystrophic changes. Intramuscular interstitial amyloid deposits are observed in approximately half of the patients. Symptomatic and supportive strategies remain the mainstay of treatment. The recent development of animal models of ANO5 muscle diseases could help achieve a better understanding of their underlying pathomechanisms and provide an invaluable resource for therapeutic discovery.

## 1. Introduction

Anoctaminopathy-5 refers to a group of clinically heterogeneous skeletal muscle or bone disorders due to mutations in *ANO5*. *ANO5* is located on chromosome 11 at 11p14.3 (OMIM 608662) and encodes anoctamin 5 (ANO5), also known as transmembrane protein 16E (TMEM16E). ANO5 is highly expressed in skeletal muscle, cardiac muscle, chondrocytes, and osteoblasts [1]. Mutations in *ANO5* were first implicated in patients with an autosomal dominant skeletal dysplastic syndrome, gnathodiaphyseal dysplasia (GDD), in 2004. The disease is characterized by bone fragility, diaphyseal sclerosis of long bones, and cemento-osseous lesions of jaw bones associated with generalized osteopenia and frequent bone fractures [1]. In 2010, biallelic mutations in *ANO5* were identified in patients with two clinically distinct forms of autosomal recessive muscular dystrophies: 1. limb–girdle muscular dystrophy type R12 (LGMDR12, or formerly known as LGMD2L) and 2. Miyoshi distal myopathy type 3 (MMD3) [2]. The phenotypic spectrum of ANO5 muscle disorders later expanded to include asymptomatic hyperCKemia and metabolic myopathy-like (pseudometabolic) phenotype (myalgia or exercise intolerance with or without rhabdomyolysis) [3,4,5]. Cardiac involvement occurs in 10–30% of patients with ANO5 muscular dystrophies, ranging from subclinical arrhythmia to symptomatic cardiomyopathy [6]. ANO5 muscle diseases and bone disorders generally do not coexist, but a combination of both phenotypes may occur on rare occasions [7,8]. To date, more than 120 mutations in *ANO5* have been identified (https://databases.lovd.nl/shared/genes/ANO5, accessed on 1 July 2022), most of which were responsible for the muscle phenotypes. 

In this review, we aimed to provide an overview of the epidemiology, clinical spectrum, histopathology, muscle imaging, animal models, and management of ANO5 muscular dystrophies. 

## 2. Epidemiology

ANO5 muscular dystrophies have a high prevalence in northern European populations, which is likely due to a founder mutation [9]. The estimated prevalence of LGMDR12 is 0.27/100,000 in the northern English population [9], 1/100,000 in the Danish population [10], and 2/100,000 in the Finnish population [11]. In northern and Central Europe, LGMDR12 is the third most common LGMD subtype following LGMDR1 and LGMDR9 [12]. It accounts for 26% of autosomal recessive LGMD (LGMDR) in the Netherlands [13] and 11% of LGMDR in Denmark. [10]. In contrast, it accounts for only about 2.6% of LGMDR in Italy (2–4 % of Italian LGMD) [14,15]. In the United States, about 13% of LGMDR cases are LGMDR12 (7 % of LGMD) [16]. ANO5 muscular dystrophies are relatively uncommon in Asian and Middle Eastern populations, with only a few reported patients from these regions [17,18,19,20,21,22]. 

## 3. Gene and Protein

### 3.1. Gene 

**Figure 1:**
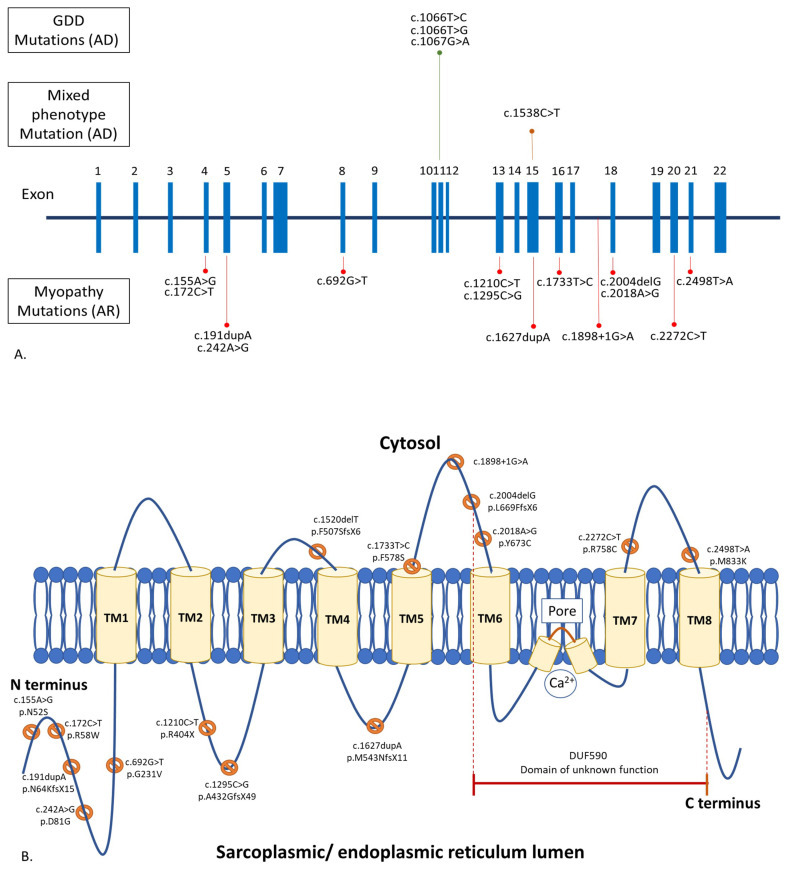
Schematic representation of anoctamin 5 gene (*ANO5*) (**A**) and protein (ANO5) (**B**) displaying the relatively common mutations underlying autosomal recessive (AR) muscle disorders (more than 5 reported cases), autosomal dominant (AD) gnathodiaphyseal dysplasia (GDD), and combined muscle and bone phenotypes.

*ANO5* contains 22 exons (Figure 1A) [23]. ANO5 muscle diseases are due to biallelic, loss-of-function mutations in *ANO5*, while GDD is a result of monoallelic, gain-of-function *ANO5* mutations. [2,12,24]. There are over 120 *ANO5* mutations linked to human diseases (https://databases.lovd.nl/shared/genes/ANO5, accessed on 1 July 2022). These mutations spread throughout the entire gene. Approximately 20% of the mutations are missense variants, and 80% are of other variant types, including nonsense, insertion, deletion, splice-site, and silent variants [12,15,25]. The most common *ANO5* mutations are c.191dupA (p.N64KfsX15) in exon 5, followed by c.1898 + 1G > A in intron 17, c.2272C > T (p.Arg758Cys) in exon 20, and c.692G > T (p.Gly231Val) in exon 8, respectively (https://databases.lovd.nl/shared/genes/ANO5, accessed on 1 July 2022). The truncating c.191dupA mutation is a founder mutation in the northern European populations [9]. Homozygosity of the c.191dupA mutation is common in England, Germany, and Denmark, ranging from 45 to 75% [9,10]. In France and the Netherlands, about 16–17% of patients carried the homozygous c.191dupA mutation [5,13]. The c.2272C > T mutation is common in Finland. Homozygosity of the c.2272C > T mutation was found in 36% of Finnish patients [11]. None of these frequent pathogenic variants were found in reported Asian patients [17,18,19,22]. There is no established genotype–phenotype correlation in anoctaminopathy-5. For example, patients carrying the homozygous c.191dupA mutation may express LGMDR12, MMD3, pseudometabolic, or asymptomatic hyperCKemia phenotype. [5,11,12,15,26]. Interfamilial and intrafamilial variabilities in clinical presentation and disease progression have also been reported [15,27].

GDD is mainly caused by the c.1066 T > C (p.Cys356Arg), c.1066T > G (p.Cys356Gly), and c.1067 G > A (p.Cys356Tyr) mutations in exon 11 of *ANO5*, with another few reported mutations in exons 7 and 15. [1,28,29,30,31,32]. 

Though the muscle and bone phenotypes of anoctaminopathy-5 do not overlap, there was a report of a large multigenerational family with combined GDD and rhabdomyolysis due to a known dominant c.1538C > T mutation in *ANO5* [8,33]. Another patient with LGMDR12, due to compound heterozygous mutations in *ANO5* (c.2018A > G and c.172C > T), was reported to have facial dysplasia and a prominent jaw without other features of GDD [7].

### 3.2. Protein

Anoctamin 5 (ANO5) is a 913-amino acid, 107 kD protein of the anoctamin family, which consists of eight transmembrane domains and one DUF590 (Domain of Unknown Function 590) or anoctamin domain (Figure 1B). It is abundantly expressed in skeletal muscle, cardiac muscle, chondrocytes, and osteoblasts [1]. ANO5 is located in intracellular vesicles and the sarcoplasmic or endoplasmic reticulum [14]. Despite its putative calcium-activated chloride channel property, the study of chloride currents in ANO5-deficient mice showed similar channel activity to their wild-type littermates [34,35]. ANO5 functions predominantly in phospholipid scrambling (PLS), which facilitates the movement of phospholipids between the membrane bilayer during various biological processes [36]. Interestingly, it was demonstrated that ANO5 is involved in a sarcolemmal repairing mechanism similar to dysferlin, in which its defects give rise to muscle diseases (dysferlinopathy), resembling anoctaminopathy-5 [37]. During the membrane repairing process, ANO5 translocates to the injured sarcolemma. Calcium and annexins are also key molecules vital to the membrane resealing process. Membrane injury leads to an influx of calcium into the cell, which then triggers the recruitment of annexins to the injured site to form a repair cap. The loss of ANO5 in a mouse model of anoctaminopathy-5 impaired the transport of annexins to the wounded sarcolemma [38]. A study in myoblasts, derived from an MMD3 patient carrying a homozygous c.2272C > T mutation, showed that the mutated protein also compromised the sarcoplasmic or endoplasmic reticulum-mediated calcium uptake, leading to prolonged injury-induced calcium transients; by lowering the cytosolic calcium level, the membrane repair defect can be restored [39]. 

Although both dysferlin and anoctamin 5 are critical to the membrane resealing process, they work in different stages of the membrane repair machinery. This was demonstrated in a mouse model of dysferlinopathy, where the overexpression of human *ANO5* did not rescue the myopathy phenotype or ameliorate the sarcolemmal repair defect [40]. 

## 4. Clinical Phenotype

ANO5 muscle diseases are more frequently seen in men, with a male:female ratio of 2:1 to 4:1 [2,9,13,14]. The relatively milder symptoms in affected females observed in several studies may, at least in part, explain this male predominance of anoctaminopathy-5 [11,12,20,41,42]; however, this sex-based difference in severity was not observed in French and Brazilian cohorts [5,26]. ANO5 muscular dystrophies are considered the diseases of early-to-mid adulthood given that the mean age of onset is between 32 and 41 years [2,5,13,43,44], but the diseases can actually affect all age groups, ranging from 4 to 85 years [26,45]. 

As mentioned earlier, biallelic mutations of *ANO5* cause a continuum of four autosomal recessive muscle phenotypes. Conversion from a pseudometabolic or asymptomatic hyperCKemia phenotype to LGMDR12 or MMD3 does occur. The frequency of each muscle phenotype of anoctaminopathy-5 varies from country to country. In general, LGMDR12 is the most common phenotype of ANO5 muscle diseases with over 300 patients reported worldwide, while approximately 50 patients with MMD3 have been reported. In a Dutch cohort, LGMDR12 accounted for 59% (38/64) of ANO5 muscle diseases, while MMD3 was 13% (8/64) [13]. In a French cohort, LGMDR12 and MMD3 accounted for only 32% (12/38) and 16% (6/38), respectively, while over half of the patients expressed asymptomatic hyperCKemia or exercise intolerance phenotype [5]. From all the reported cases, the frequencies of LGMDR12, MMD3, pseudometabolic phenotype, and asymptomatic hyperCKemia are 66%, 11%, 11%, and 11%, respectively [2,3,4,5,6,9,10,11,12,13,14,15,16,17,18,19,20,21,22,25,26,27,29,37,41,44,45,46,47,48,49,50,51,52,53,54,55,56,57,58,59]. 

### 4.1. LGMDR12

**Figure 2:**
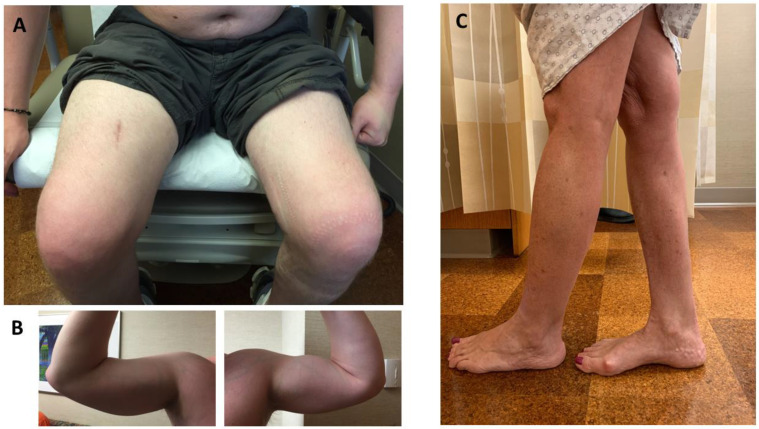
Clinical findings of a patient with ANO5 limb–girdle muscular dystrophy (LGMDR12) and recurrent rhabdomyolysis (**A**,**B**) and a patient with ANO5 distal myopathy of Miyoshi type (MMD3) (**C**). (**A**). Asymmetric atrophy of quadriceps. (**B**). Asymmetric atrophy of biceps brachii. (**C**). Asymmetric atrophy of gastrocnemius.

Patients typically develop pelvic girdle weakness followed by an involvement of the shoulder girdle musculature. Asymmetric weakness and atrophy are common (up to 90% of patients), leading to a classic feature of asymmetrical quadriceps femoris and biceps brachii atrophy (Figure 2A,B) [9,13]. Despite LGMDR12 being considered a proximal myopathy, calf atrophy or pseudohypertrophy is a common finding on the exam [2,9,14,19,26,27,41,50]. Gastrocsoleus weakness may occur, but to a lesser degree compared to MMD3 patients; however, it can become more evident in the advanced stage of LGMDR12. Tibialis anterior involvement leading to foot drop is uncommon [21]. Axial muscle weakness has been reported in up to 36% (9/25) of patients [4,12,26], and rarely it could be the sole manifestation of the disease [26]. Mild dysphagia has been reported in 16–46% of patients [10,26]. About 20–35% of patients may have scapular winging. Mild joint contractures were observed in 10–38% of patients [9,10,12]. 

The common initial symptoms are difficulties walking long distances or walking uphill/upstairs [9,41]. Some may present with myalgia, cramps, or muscle stiffness [14,19,26]. Other initial presentations include exercise intolerance, asymmetric muscle atrophy without weakness, or rhabdomyolysis [4,55]. Age at symptom onset varies from 12 to 85 years (median 38 years) [13,45]. LGMDR12 is generally considered a slowly progressive disease given that most patients maintain the ability to walk for several decades after the symptom onset; however, a more rapidly progressive course of weakness leading to ambulatory loss within 10 years has also been reported [9,10]. Less than 10% of LGMDR12 patients require gait aids or wheelchairs. The median age at ambulatory loss is 58 years [9,10,13,26,43,51,54]. Asymmetric weakness or atrophy and later onset help differentiate LGMDR12 from LGMDR2 (LGMD2B) [9]. Creatinine kinase (CK) levels are typically very high with an average of 4500 U/L, but can range from 500 to 32,000 U/L [9,15,26]. HyperCKemia can precede the onset of weakness by up to 26 years [4,19]. The degree of CK elevation does not always correlate with the symptom severity [11]. 

### 4.2. MMD3

Patients initially develop asymmetric weakness and atrophy of calf muscles (Figure 2C). Calf atrophy usually precedes overt calf weakness, which can be up to 15 years in some patients [18,47]. On the exam, calf enlargement is very common in the early stage of the disease, but eventually, the calves become atrophic as the disease progresses. [27,50]. In the later stage, some may experience hip, followed by shoulder girdle, weakness [2]. Hand weakness is uncommon [12]. Weakness can be symmetrical, albeit rare [48]. Compared to the LGMDR12 phenotype, proximal musculature is less affected clinically, but as the disease progresses, these two phenotypes can converge [9]. Some patients may have minor contractures [10]. Mild dysphagia has also been reported in one patient [10]. Another patient was reported with hypoplastic genitalia [18].

The common initial symptoms are difficulties walking on tiptoes or calf atrophy [2,9,47]. Abnormal sensations such as burning or tightness in the calves have also been reported [27]. Some patients may have an asymptomatic elevation of CK levels long before the onset of weakness [47]. Age at onset ranges from 15 to 51 years (mean at 33 years) [47,50,51]. Disease is slowly progressive. Only a few patients needed gait aids decades after the disease onset [10,27]. CK levels are typically very high, ranging from 700 to 35,000 U/L (2) [11,27,51].

In comparison to dysferlin-deficient Miyoshi myopathy or Miyoshi distal myopathy type 1 (MMD1), MMD1 patients typically have more symmetrical involvement and a younger age of onset (usually before 30 years old) [27,47]. 

### 4.3. Pseudometabolic Phenotype

Myalgia, exercise intolerance, and/or rhabdomyolysis without muscle weakness are the key clinical features of patients in this group. [3,4,22,26,51]. These symptoms mimic the classic manifestations of metabolic myopathies, hence the term pseudometabolic phenotype. This particular phenotype was initially thought to be more prevalent in females [11]; however, subsequent studies showed an equal distribution of this phenotype in males and females [3,5,11,12,15,22,26,51,59]. Symptom onset varies from 4 to 75 years [15,26]. A proportion of these patients subsequently convert to the LGMDR12 or MMD3 phenotype [3,5,20,41]. With a median follow-up period of 5 years, 83% (10/12) of patients with myalgia remained stable without clinical weakness [5]. CK levels range from 200 to over 47,000 U/L [5,26], and some patients may have hyperCKemia long before being symptomatic [22]. Of note, rhabdomyolysis can also occur in anoctaminopathy-5 patients with overt weakness [3,6].

### 4.4. Asymptomatic HyperCKemia

The age of reported first detection of hyperCKemia varies from 11 to 69 years with CK levels ranging from 500 to 40,000 U/L [5,15,26,44]. A neurologic exam may reveal calf pseudohypertrophy in some patients [44]. As mentioned earlier, a proportion of patients with other ANO5 muscle phenotypes may carry a diagnosis of asymptomatic hyperCKemia predating the onset of symptoms. There is no known predicting factor for phenotypic conversion in anoctaminopathy-5 [44]. 

A carrier of the c.191dupA mutation has been reported to have asymptomatic hyperCKemia [3]. Another carrier of the c.1640G > A mutation had muscle cramps and mild CK elevation with mild myopathic changes observed in a muscle biopsy [41]. 

## 5. Cardiorespiratory Involvement in ANO5 Muscular Dystrophies

As mentioned earlier, ANO5 is also highly expressed in cardiac muscle. Cardiac involvement occurs in all subtypes of ANO5 muscle disorders [4,5,10,13,51,60]. It was initially thought that these ANO5 muscle diseases did not affect the life expectancy of patients, owing to the lack of significant cardiorespiratory involvement; however, subsequent series of anoctaminopathy-5 patients with catastrophic cardiac diseases have been reported. About 30% of patients have cardiac arrhythmia, in which frequent premature ventricular contractions (PVCs) are the most common type of arrhythmia [10]. Other reported cardiac arrhythmias include sinus bradycardia, paroxysmal atrial fibrillation, supraventricular tachycardia, left anterior fascicular block, and a short PQ interval [4,15,51]. Left ventricular involvement has been reported in 10–30% of patients, including left ventricular dysfunction (ejection fraction < 45%), left ventricular dilatation, and dilated cardiomyopathy [13,51]. Hypertrophic cardiomyopathy and asymptomatic intraventricular septum thickening are rare, with only two reported cases of each [26,41]. Septal hypokinesia is also uncommon [4]. 

Respiratory muscle involvement is infrequent and typically mild. Abnormal findings include mildly reduced maximal voluntary ventilation, maximal respiratory pressures, and vital capacity [6,26,55]. Only a small proportion of patients reported dyspnea [20], and ventilatory support was necessary in very rare circumstances [13,15,26]. 

## 6. Myopathology of ANO5 Muscle Diseases

Muscle biopsy may display a broad array of histopathological findings, ranging from normal to severe dystrophic changes (marked variation in fiber size, necrotic and regenerating fibers, and endomysial fibrosis) [5,9,22]. The severity of myopathic changes does not necessarily correlate with the degree of muscle weakness, as dystrophic pathology can be seen in up to 50% of patients with asymptomatic hyperCKemia [5]. Lobulated fibers, whorled fibers, ring fibers, pyknotic nuclear clumps, and rimmed vacuoles have been described in some patients [4,9,21,22,57]. 

Inflammatory infiltrates were reported in 13 to 31% of patients [9,61], ranging from scattered small foci of inflammatory infiltrates to prominent inflammatory exudates with, or without, autoaggressive features, mimicking inflammatory myopathies [9,11,14,20,21,44,59]. The perivascular cuffing of histiocytes or necrotizing vasculitis pathology have been rarely observed [4,49]. Other unusual myopathological findings include the presence of numerous ragged-red or ragged-red-like fibers or a mild increase of lipid droplets [45,59]. 

**Figure 3:**
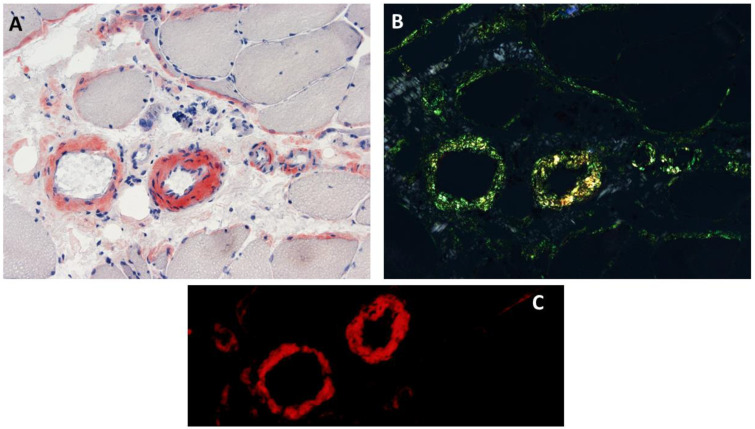
Histopathological findings in ANO5 muscle diseases. Congophilic deposits in intramuscular blood vessels and perimysium adjacent to perifascicular fibers are observed on Congo red stained section viewed under bright light (**A**), polarized light, (**B**) and rhodamine optics (**C**).

Congophilic deposits within intramuscular blood vessels and/or connective tissue elements (endomysium or perimysium) were reported in 53% (8/15) of patients with ANO5 muscle disease (Figure 3), who had no evidence of systemic amyloidosis [6]. Although there is no genotype–phenotype correlation regarding the interstitial amyloid deposition in anoctaminopathy-5, intramuscular interstitial amyloidosis has been reported in the patients homozygous for the c.191dupA mutation [6]. Therefore, the frequency of intramuscular interstitial amyloidosis could be higher in an anoctaminopathy-5 cohort with a high percentage of c.191dupA homozygosity. This interstitial amyloidosis observed in ANO5 muscular dystrophies is similar to what is observed in some patients with dysferlinopathy [3,4,6,20,22,25,49]. Isolated amyloid myopathies (IAMs) refer to a group of genetically heterogenous myopathies featuring amyloid deposits restricted to skeletal muscles, without any evidence of systemic amyloid deposition. ANO5 muscular dystrophies are the most common cause of IAMs, accounting for 57% (8/14) of patients, followed by dysferlinopathy (2/14 or 14%) [56,62]. The mass-spectrometry-based proteomic analysis of intramuscular congophilic deposits obtained from ANO5 muscular dystrophy patients confirmed the presence of amyloid chaperone proteins (e.g., serum amyloid P and/or apolipoprotein E) but failed to identify ANO5 within the deposits [4]. The clinical significance of localized amyloidosis affecting skeletal muscle in anoctaminopathy-5 patients remains unknown. In a cohort of 15 patients with ANO5 muscular dystrophies, there was no significant difference of either muscle or cardiac phenotypes between patients with, and without, interstitial amyloidosis [6]. 

A Western blot study using monoclonal antibody clone N421A/85 (UC Davis/NIH NeuroMab, Davis, CA, USA) was able to show a reduction in ANO5 expression in muscle lysates obtained from selected patients with ANO5 muscle diseases [58]. The immunohistochemical study of ANO5 had not been successful until recently, possibly due to its low immunogenicity and hydrophobic nature [58]. Using the same monoclonal antibody clone N421A/85, Xu and colleagues showed the absence of ANO5 immunoreactivity in frozen muscle obtained from a patient carrying a homozygous c.191dupA mutation, while sarcoplasmic aggregates overreactive for ANO5 were observed in a patient carrying c.191dupA and c.363 + 4A > G mutations [63]. Immunohistochemical and Western blot studies of dystrophin and calpain-3 yielded variable results. Secondary dystrophin and/or calpain 3 deficiency was observed in Western blot studies in 54% (7/13) of patients [11]. The immunohistochemical study of dysferlin revealed the marked depletion of sarcolemmal immunoreactivity in some patients and normal in others [9,11,14,18,48,49]. 

With electron microscopy, basal lamina duplication with separation from sarcoplasm and multifocal sarcolemmal membrane disruption without subsarcolemmal vesicle accumulation can be seen [14,27].

## 7. Electrophysiologic Study

In symptomatic patients, EMG generally shows short-duration, low-amplitude motor unit potentials with early recruitment, and fibrillation potentials are frequently observed [4,5]. Long-duration and high-amplitude motor unit potentials may be present, which indicate the chronicity of an underlying myopathic process [64]. In asymptomatic hyperCKemia patients, EMG is normal in 65% of patients [44]. Three patients with LGMDR12 have been reported to have myotonic discharges (two in the proximal muscles and one in the gastrocnemius) without associated clinical myotonia [4]. 

## 8. Muscle MRI

**Figure 4:**
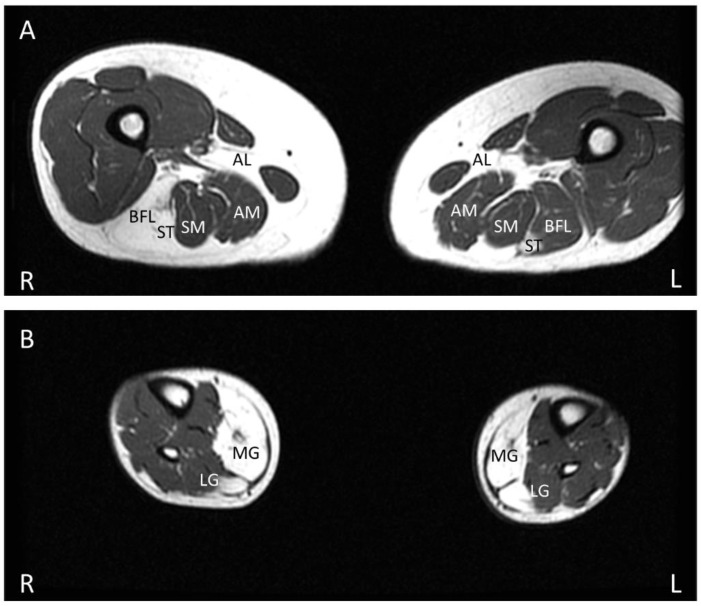
T1-weighted lower limb MRI in a patient with LGMDR12, calf atrophy and longstanding history of exercise intolerance and rhabdomyolysis showing asymmetrical fatty replacement of predominantly posterior lower limb compartment muscles, including bilateral adductor longus, long head of right biceps femoris, and bilateral semitendinosus (**A**) and bilateral medial gastrocnemius and parts of lateral gastrocnemius (**B**). AL—adductor longus, AM—adductor magnus, SM—semimembranosus, ST—semitendinosus, BFL—long head of biceps femoris, MG—medial gastrocnemius, LG—lateral gastrocnemius.

In the initial imaging study of MMD3 patients, the fibrofatty replacement of muscle tissue, as evident by an increase in T1 intensity in muscle, was observed initially and predominantly in distal lower limb musculature, followed by an involvement of proximal musculature as the disease progressed [50]. However, subsequent studies found no correlation between the pattern of affected muscles on MRI and the clinical phenotypes [26,52]. In both LGMDR12 and MMD3 phenotypes, asymmetric muscle involvement with variable fatty replacement is typically prominent in biceps brachii and the posterior compartment musculature of lower limbs (Figure 4A,B), including the adductor magnus, adductor longus, semimembranosus, semitendinosus, long head of biceps femoris, gastrocnemius (medialis more than lateralis), and soleus [9,17,27,42,44,65,66]. Compared to these muscles, the quadriceps femoris is less severely replaced by fatty connective tissue in the early stage of the disease. Fatty replacement is more prominent in the distal portion of the quadriceps compared to its proximal region [26]. The rectus femoris is generally the least affected component of the quadriceps [11,52]. A central shadow in the rectus femoris and an undulating fascia sign with a wavy fascia between the severe fatty infiltrated vastus intermedius and vastus lateralis muscles can be seen [26,52]. In many hereditary myopathies, the gracilis and sartorius are generally spared, even at the late stage of the diseases, but these two muscles can be affected in the advanced stage of anoctaminopathy-5 [9,42,50,67]. Anterior and lateral distal lower limb muscles are typically spared even at the very late stage of ANO5 muscle diseases [9,42,50,52]. 

This fatty infiltration of muscle tissue generally precedes the onset of weakness. The fatty replacement of calf muscles was observed on muscle MRI in 56% of patients who presented with exercise intolerance or asymptomatic hyperCKemia [5]. As mentioned earlier, some studies showed that females generally have milder phenotypes compared to males. A recent muscle MRI study also showed milder muscle atrophy and fatty replacement in females than in males [42].

On the Short Tau Inversion Recovery (STIR) sequence of MRI, there was no significant difference in myoedema patterns when compared between anoctaminopathy-5 patients with and without weakness [61]. 

The aforementioned MRI findings are nonspecific, and it is difficult to differentiate LGMDR12 from other recessive LGMDs by solely comparing MRI findings [52]. Dysferlinopathy (LGMDR2) has fairly similar muscle MRI findings to those of anoctaminopathy-5 patients, but anoctaminopathy-5 patients tend to have more asymmetrical muscle involvement, more gluteus minimus and gluteus medius involvement, and a spared tibialis anterior even in the later stage of the disease [50,52,68]. 

## 9. Animal Models

Two initial mouse models of anoctaminopathy-5 that were created by the complete disruption of *Ano5* failed to show significant clinical myopathy or cardiomyopathy [69,70]. Later on, mouse models achieved by truncated ANO5 expression showed a myopathic phenotype, including variable degrees of weakness and muscle atrophy, exercise intolerance, and myofiber injury, as a result of defective membrane repair machinery [35,36]. The transduction of AAV-*Ano5* into the knock-out mice rescued the membrane repair defect [36]. A rabbit model, developed by a CRISPR-engineered technique to insert indels into exon 12 and/or 13 leading to a frame-disrupting ANO5 mutation, resulted in ANO5 loss-of-function and resembled the human muscular dystrophy phenotype with dystrophic changes observed in the gastrocnemius, tibialis anterior, tongue, and diaphragm [71]. These animal models provide invaluable help to understand the pathomechanism of anoctaminopathy-5 and to test the pre-clinical safety and efficacy of novel therapeutic strategies, such as gene therapy. 

## 10. Management

Currently, there is no specific treatment or cure for ANO5 muscular dystrophies. Symptomatic and supportive strategies remain the mainstay of treatment. The weakness and fear of further muscle damage from physical activities may limit patients from exercise and predispose them to a sedentary lifestyle. Aerobic exercise with moderate intensity on a cycle ergometer for 10 weeks has been demonstrated to improve cardiovascular fitness, with an increase in VO_2max_ (maximum rate of oxygen consumption) up to 27% and an improvement in functional ability by decreasing time in repetitive sit-to-stand without deleterious effects on muscle strength, muscle pain, or CK levels. There was also some improvement in plantar flexion strength [54]. Therefore, exercise is beneficial for patients with ANO5 muscle disorders, but should be performed under professional supervision to avoid an overexertion that may trigger rhabdomyolysis [55].

All patients with muscular anoctaminopathy-5 should undergo cardiac evaluations for the surveillance of any cardiac contractility and conduction defect [5]. Patients with respiratory symptoms should also be screened by a pulmonary function test and/or an overnight oximetry. Treatment with ACE inhibitors in anoctaminopathy-5 patients with left ventricular dysfunction has been suggested [51].

## 11. Conclusions

Anoctaminopathy-5 is a common cause of autosomal recessive muscular dystrophies in northern Europe due to a founder mutation, although they have also been reported in other parts of the world. Biallelic mutations in *ANO5* disrupt the sarcolemma repair machinery and give rise to a continuum of four muscle phenotypes: LGMDR12, MMD3, pseudometabolic phenotype, and asymptomatic hyperCKemia. When muscle weakness is present, asymmetry is common. Cardiac involvement occurs in 10–30% of patients, including those without overt muscle weakness, while severe respiratory insufficiency is rare. The interstitial amyloidosis of skeletal muscle is a unique histopathological finding observed in approximately half of the patients; however, its significance remains to be further elucidated. Currently, there is no effective disease-modifying agent for ANO5 muscle diseases. The recent development of animal models of anoctaminopathy-5 could help achieve a better understanding of its underlying pathomechanism and provide an invaluable resource for therapeutic discovery. 

## Data Availability

All relevant data have been cited within this review article.
